# Devoured AI benevolence? How and when AI benevolence triggers employees’ taking charge within the zero-sum jungle

**DOI:** 10.3389/fpsyg.2026.1931407

**Published:** 2026-10-14

**Authors:** Bin Zhang, Yanlin Ma, Xiaolei Qian

**Affiliations:** 1 Business School, Sichuan University, Chengdu, China; 2 College of Finance and Economics, Sichuan International Studies University, Chongqing, China; 3 ELM Graduate School, HELP University, Kuala Lumpur, Malaysia

**Keywords:** AI benevolence, human-AI interaction, metacognitive strategies, taking charge, zero-sum mindset

## Abstract

Employees increasingly perceive generative artificial intelligence (GenAI) as a supportive and benevolent workplace entity. Yet, despite examining the disruptive challenges and broad benefits of AI, extant organizational literature fails to adequately explicate the higher-order cognitive mechanisms through which AI benevolence fosters proactive, change-oriented employee behavior. Drawing on cognitive appraisal theory (CAT), this study examines the mechanisms and boundary conditions through which AI benevolence fosters employees’ taking-charge behavior. Results from a three-wave survey of 313 internet industry employees demonstrate that: (i) metacognitive strategies mediated the relationship between AI benevolence and individuals’ taking-charge behavior; (ii) the employee’s mindset attenuated the positive effect of AI benevolence on metacognitive strategies, consequently dampening the overall indirect effect on taking-charge behavior. Ultimately, this research contributes to organizational behavior literature by elucidating how GenAI shapes employee proactivity in AI-augmented work environments.

## 1 Introduction

As over 72% of global enterprises integrate generative artificial intelligence (GenAI) into their core workflows (McKinsey & Company, 2024), the workplace is undergoing a profound transformation. The disruptive nature of this shift lies not merely in enhanced computational power, but in a fundamental change in how employees relate to technology (Kumar et al., 2025; Agarwal et al., 2026). Modern AI systems are increasingly designed to exhibit a specific, positive relational intent: AI benevolence. Platforms like Copilot, for instance, are positioned not just as capable assistants, but as non-judgmental “virtual colleagues” offering error tolerance and unconditional support. Consequently, routine interactions with AI have transcended mechanical command-and-execution cycles to include meaningful affective exchanges (Karpus et al., 2021; Dennis et al., 2023). Distinct from mere AI warmth or empathy (which primarily focus on friendliness or the ability to recognize emotions), we conceptualize AI benevolence as an actionable goodwill. From the employee’s perspective, AI benevolence captures the extent to which employees believe the AI system acts as a non-judgmental, supportive ally that has their best interests at heart, rather than functioning as an instrument for managerial surveillance or solely maximizing corporate profits (Shi et al., 2021; Dennis et al., 2023). Yet this benevolence introduces a critical, unresolved managerial dilemma: Does a highly benevolent, non-judgmental AI lull employees into complacency, or does it provide the necessary psychological resources to empower them to challenge the status quo and engage in proactive behaviors such as taking charge?

Recent literature on human-AI collaboration increasingly recognizes AI’s enabling role, exploring its positive impact on employee outcomes (Einola and Khoreva, 2023; Ma et al., 2026). However, a critical gap remains: while previous studies have largely focused on the instrumental or functional utility of AI, they have underexplored the relational and affective dimensions of human-AI interactions. Although the computers-are-social-actors (CASA) paradigm suggests that individuals instinctively generate social responses to the affective cues of technology, current literature largely neglects how AI’s emotional expressions, particularly its benevolence as a relational agent, reshape employees’ foundational cognitive appraisals of their work environment (De Kervenoael et al., 2024). First, prior studies have not adequately examined whether non-judgmental tolerance and goodwill (i.e., AI benevolence) provide employees with the psychological resources needed to challenge the status quo and reconfigure workflows (Lin et al., 2024; Dong et al., 2026). Consequently, it remains unclear how such benevolence promotes high-risk, proactive, change-oriented behaviors, such as taking charge, defined as voluntary and constructive efforts to effect organizationally functional change (Parker and Collins, 2010). Second, existing research has not sufficiently identified the higher-order cognitive mechanisms through which AI benevolence translates into proactive behaviors. The literature is frequently dominated by discussions of whether AI reliance leads to cognitive inertia or deskilling, thereby neglecting its potential to foster higher-order cognitive development (Dennis et al., 2023; Zhang et al., 2026). This perspective overlooks a crucial theoretical possibility: by alleviating lower-order anxieties and freeing cognitive bandwidth, AI benevolence may serve as a catalyst for activating metacognitive strategies (i.e., the self-monitoring, reflection, and regulation of one’s cognitive processes). Activating this higher-order cognition is likely critical for explaining how AI benevolence elevates the human role and triggers proactive change in an AI-augmented era. Finally, in delineating the boundary conditions of human-AI collaboration, extant research primarily focuses on the immediate interaction, often overlooking the constraining influence of employees’ individual contextual beliefs, such as a zero-sum mindset (Jin et al., 2025; Paek and Kakkar, 2025). Specifically, it remains unclear how a cue conflict, arising when AI benevolence interacts with this individual zero-sum mindset, alters employees’ cognitive appraisals and subsequent behaviors.

To address these theoretical gaps, this study draws upon CAT to investigate the mechanisms and boundary conditions governing how and when AI benevolence fosters employees’ taking charge behaviors. According to CAT, individuals process environmental stimuli through primary appraisals (evaluating potential threats or benefits) and secondary appraisals (evaluating coping potential and strategies) (Folkman et al., 1986; Ellsworth, 1991; Dong et al., 2026). We posit that AI benevolence alters employees’ primary appraisals by reframing the AI from a potential competitor into a supportive resource, thereby mitigating defensive resource depletion and freeing cognitive and affective resources (Folkman et al., 1986; Xu et al., 2026). Consequently, these liberated resources empower employees to mobilize higher-order metacognitive strategies during secondary appraisals. As AI absorbs routine tasks, employees with enhanced psychological security leverage their freed cognitive bandwidth to critically reflect on existing workflows and strategies (De Boeck and Liu, 2026; Dong et al., 2026). This metacognitive engagement enables them to identify unique human value within human-AI collaborative ecosystems. Ultimately, such reflection motivates and equips employees to pinpoint procedural inefficiencies, catalyzing their willingness to break from convention and take charge (Ahmad et al., 2025; Qiao et al., 2026). Furthermore, recognizing that employees’ reactions to AI are fundamentally shaped by how they perceive resource availability, we introduce an individual’s zero-sum mindset as a critical boundary condition (Ellsworth, 1991; Dong et al., 2026). This study further posits that a high zero-sum mindset disrupts individuals’ primary appraisals of AI benevolence. When viewed through this hypercompetitive cognitive lens, the benevolent expressions of an AI are susceptible to being interpreted as managerial hypocrisy or manipulative tactics designed to lower employees’ vigilance (Jin et al., 2025; Paek and Kakkar, 2025). Consequently, this cognitive interference attenuates the release of cognitive resources and the subsequent activation of metacognitive strategies.

Drawing on CAT, this study theorizes and empirically investigates how and when AI benevolence fosters employees’ taking-charge behavior, contributing to the organizational behavior and human-AI interaction literatures. First, we advance human-AI interaction research by elucidating how AI benevolence empowers employees to challenge the status quo. Although recent scholarship recognizes the broad benefits of AI integration, how specific AI characteristics, such as non-judgmental tolerance and goodwill, provide the psychological resources necessary for high-risk, proactive behavior remains underexplored (Lin et al., 2024; Dong et al., 2026). By linking AI benevolence to taking-charge behavior, this study shifts the theoretical conversation beyond basic task facilitation, demonstrating how AI catalyzes voluntary, constructive, and change-oriented organizational efforts. Second, by establishing the mediating role of metacognitive strategies, our research broadens the application of CAT. Prior inquiries into the behavioral consequences of AI have predominantly focused on lower-order affective responses (e.g., satisfaction, trust) or task-based metrics (e.g., workload reduction), leaving higher-order cognitive mechanisms underexplored (Dennis et al., 2023; Dong et al., 2026). Our findings elucidate how freed cognitive bandwidth is repurposed for higher-order reflection, thus enriching the secondary appraisal mechanisms within CAT. Finally, we expand the theoretical boundaries of CAT by introducing employee zero-sum mindset as a critical boundary condition. Extant research primarily focuses on the immediate interaction, often overlooking the constraining influence of employees’ individual contextual beliefs (Jin et al., 2025; Paek and Kakkar, 2025). By unpacking how this cognitive interference, arising from a cue conflict between AI benevolence and a zero-sum mindset, neutralizes the positive effects of AI, this study provides a more nuanced understanding of how individual differences shape human-AI dynamics.

## 2 Theory and hypothesis development

### 2.1 AI benevolence and metacognitive strategies

Metacognitive strategies involve the self-monitoring, reflection, and regulation of one’s cognitive processes (i.e., thinking about thinking) (Sun et al., 2025; Ali et al., 2026). Typical manifestations include proactively assessing work method efficacy, reflecting on decision-making rationality, and recalibrating task objectives. As higher-order cognitive activities, these strategies are resource-intensive, demanding substantial cognitive bandwidth and psychological capital (Ali et al., 2026). While adjacent constructs like anthropomorphism, AI empathy, and supportiveness merely describe the system’s human-like form, emotion-recognition capabilities, or functional utility, they do not guarantee a benign moral intent (Alabed et al., 2022; Liu et al., 2024). Distinct from general warmth or global trustworthiness, AI benevolence uniquely isolates this underlying motivation, reflecting the extent to which the AI acts as a non-judgmental, protective ally prioritizing the employee’s best interests (Shi et al., 2021; Dennis et al., 2023). Drawing on CAT, we posit that AI benevolence reshapes employees’ cognitive appraisals of their work environment, thereby providing the psychological resources and motivation necessary to engage in metacognitive strategies (Folkman et al., 1986; Ellsworth, 1991; Dong et al., 2026).

Specifically, individuals undergo a primary appraisal to assess whether external stimuli are beneficial, irrelevant, or stressful to their well-being. In contemporary digital workplaces, the integration of advanced AI can initially introduce uncertainty and cognitive load as employees navigate unfamiliar human-machine dynamics (Shi et al., 2021; Kumar et al., 2025). However, when AI manifests benevolence, distinctly characterized by non-judgmental interactions, unconditional task support, and error-tolerant feedback rather than mere superficial friendliness or empathy, employees tend to appraise the technology as a reliable, supportive job resource (Shi et al., 2021; Xu et al., 2026). This benign primary appraisal significantly mitigates evaluation apprehension and the fear of failure. It establishes a baseline of psychological safety, providing employees with the “cognitive slack” required for higher-order cognitive endeavors (Gabriel et al., 2022; Dennis et al., 2023). Subsequently, during secondary appraisal, individuals evaluate available coping strategies to address their current work demands (Folkman et al., 1986; Dong et al., 2026). Within the context of human-AI collaboration, recognizing that a benevolent AI system patiently handles routine tasks and tolerates mistakes without judgment, employees realize they are freed from the constraints of low-level execution. The psychological safety engendered by this actionable goodwill alleviates the anxiety of trial-and-error (Shi et al., 2021; Dennis et al., 2023). Consequently, employees are psychologically empowered to invest their liberated cognitive bandwidth into metacognitive activities. By transcending specific executional details, employees can monitor workflows holistically, reflect on the efficiency of human-AI collaboration, and continuously recalibrate their strategies, ultimately achieving optimal complementarity between human and artificial intelligence (De Boeck and Liu, 2026).

*H1*: AI Benevolence was positively related to individuals’ metacognitive strategies.

### 2.2 The mediating role of metacognitive strategies

Taking charge behavior refers to employees’ self-initiated and constructive efforts to challenge the status quo, aimed at instigating systemic improvements to organizational workflows, policies, or services (Parker and Collins, 2010; Qiao et al., 2026). Distinct from general organizational citizenship behaviors (e.g., helping colleagues), taking charge is inherently disruptive and socially risky, as it entails altering established organizational routines (McAllister et al., 2007; Parker and Collins, 2010; Qiao et al., 2026). We posit that metacognitive strategies, serving as higher-order processes of self-monitoring and regulation, equip employees with the cognitive insight and behavioral agency required to enact such high-risk behaviors. Specifically, metacognitive strategies first provide employees with the insight necessary to identify systemic issues and opportunities for change (McAllister et al., 2007; Zahoor et al., 2024). Elevated levels of metacognition prompt individuals to transcend granular task details, monitor workflows from a macro-level perspective, and critically reflect on existing strategies. Through sustained cognitive self-monitoring, employees become significantly more adept at detecting inefficiencies and rigidities within the work system, including frictions embedded in human-AI collaboration (Sun et al., 2025; De Boeck and Liu, 2026). Furthermore, following secondary appraisal, metacognitive strategies facilitate problem-focused proactive coping (Ahmad et al., 2025; Dong et al., 2026). When employees uncover latent problems and formulate viable solutions through deliberate reflection, they cultivate a heightened sense of self-efficacy and environmental mastery. Against the backdrop of human-AI complementarity, employees recognize that implementing these reflective insights via taking charge is essential to fundamentally reshaping the work system and solidifying their unique occupational value (Zahoor et al., 2024; Qiao et al., 2026). Accordingly, we propose the following:

*H2*: Metacognitive strategies were positively related to taking charge.

Synthesizing the arguments from H1 and H2, we propose that metacognitive strategies mediate the relationship between AI benevolence and taking charge behavior. Grounded in CAT’s framework of environmental stimuli, cognitive processing, and behavioral output, we argue that AI benevolence activates employees’ metacognitive strategies, which subsequently drive taking charge behaviors (Folkman et al., 1986; Dong et al., 2026). Specifically, AI benevolence serves as a positive environmental cue. During primary appraisal, this cue prompts individuals to lower their defensive barriers against technology, thereby liberating cognitive bandwidth. During subsequent secondary appraisal, the psychological safety derived from a reduction in evaluative and displacement threats enables employees to address the role identity challenges inherent in human-AI collaboration (Shi et al., 2021; Xu et al., 2026). Consequently, individuals are empowered to channel their liberated cognitive and emotional resources into resource-intensive metacognitive strategies. Furthermore, these metacognitive strategies enable employees to detect frictions between existing organizational routines and AI systems (i.e., problem identification) and facilitate the conceptualization of systemic improvements through deliberate planning (i.e., problem-solving) (Zahoor et al., 2024; Ahmad et al., 2025; He et al., 2026). In essence, AI benevolence stimulates metacognitive processing by providing the psychological safety and resources necessary to initiate change. This manifests as critical insight into systemic flaws and the behavioral agency required to take action. Since individuals utilizing metacognitive strategies tend to adopt problem-focused coping mechanisms, they are significantly more likely to exhibit taking charge behaviors aimed at reconfiguring the broader work system (Dong et al., 2026; Qiao et al., 2026).

*H3*: Metacognitive strategies mediated the effect of AI Benevolence on taking charge.

### 2.3 The moderating role of zero-sum mindset

CAT emphasized that individuals’ cognitive processing of specific stimuli does not occur in a vacuum; rather, it is highly contingent upon contextual environmental cues (Ellsworth, 1991; Dong et al., 2026). We propose that zero-sum mindset, defined as the prevalent belief among employees that organizational resources (e.g., promotion opportunities and bonuses) are strictly finite, such that one’s success invariably necessitates another’s loss, serves as a critical boundary condition shaping the relationship between AI benevolence and cognitive processing (Jin et al., 2025; Paek and Kakkar, 2025). Specifically, an individual’s zero-sum mindset moderates the positive relationship between AI benevolence and metacognitive strategies. During the primary appraisal phase, employees harboring a high zero-sum mindset evaluate workplace interactions through a hypercompetitive cognitive lens (Jin et al., 2025; Xu et al., 2026). For these individuals, their deep belief that resources are strictly finite effectively overrides the localized benevolence experienced during human-AI interactions. Because operating under such a scarcity schema induces a chronic defensive state, these employees are predisposed to view any emerging technologies with skepticism. Consequently, the benevolence exhibited by AI introduces a significant cue conflict within individuals’ cognitive systems. Consistent with CAT, since individuals guided by a zero-sum mindset harbor a highly defensive mental model, their primary appraisals tend to prioritize threat cues (Paek and Kakkar, 2025; Dong et al., 2026). Thus, even if the AI itself poses no direct harm, these employees deplete their limited cognitive bandwidth by guarding against perceived peer competition and anticipated resource loss, thereby neutralizing the AI benevolence’s potential to activate metacognitive strategies.

More critically, a higher zero-sum mindset may induce individuals to perceive AI benevolence as digital hypocrisy, a manipulative tactic designed to lower their vigilance and extract greater labor value (Zhu and Li, 2025; De Boeck and Liu, 2026). This deep-seated individual cynicism exhausts employees’ cognitive resources. Lacking cognitive slack, employees tend to rely on lower-order, reflexive task execution merely to protect their immediate interests. Under such cognitive constraints, they lack both the capacity and motivation to initiate resource-intensive, higher-order metacognitive strategies (Xu et al., 2026; Paek and Kakkar, 2025). Conversely, when employees possess a low zero-sum mindset, there is high cognitive congruence between their internal cooperative schemas and the AI’s benevolent cues. As these individuals do not inherently view their workplace through a lens of cutthroat competition, they are not compelled to expend substantial mental resources guarding against peers (Paek and Kakkar, 2025). In this receptive cognitive state, AI benevolence is readily validated during primary appraisal as a genuine, supportive resource. Without the interference of survival anxiety, this cognitive alignment effectively mitigates employees’ psychological defenses and liberates ample cognitive bandwidth (Dong et al., 2023; De Boeck and Liu, 2026). Fortified by these preserved cognitive resources, employees can smoothly transition to secondary appraisal, channeling their energies into metacognitive activities such as reflecting on human-AI synergistic efficiency and conceptualizing novel work paradigms.

*H4*: Zero-sum mindset moderated the relationship between AI benevolence and metacognitive strategies, such that the relationship is weaker at higher (vs. lower) levels of Zero-sum mindset.

### 2.4 The moderated mediating effect

Synthesizing the arguments from H1-H4, we posit a moderated mediation model wherein a zero-sum mindset moderates the mediating effect of metacognitive strategies. According to CAT, an individual’s coping behavior is the behavioral outcome of their primary and secondary appraisals of environmental stimuli (Folkman et al., 1986; Dong et al., 2026). When cognitive processing during these appraisal stages is constrained by contextual factors, downstream behaviors are subsequently inhibited. Therefore, we propose that a high zero-sum mindset attenuates the mediating role of metacognitive strategies in the relationship between AI benevolence and taking charge behavior. For individuals with a higher zero-sum mindset, an internally hypercompetitive schema ensures that the positive cues of AI benevolence are overshadowed by ingrained concerns over resource competition. Preoccupied with guarding against perceived threats, these employees experience continuous cognitive depletion due to chronic defensive monitoring, leaving them with insufficient cognitive resources to engage in metacognitive strategies (Jin et al., 2025; Xu et al., 2026). This deficit in metacognitive processing, in turn, deprives individuals of the insight needed to identify operational flaws and the behavioral agency required to take proactive action. Consequently, regardless of how benevolent the AI appears, these employees are predisposed to adopt withdrawal or defensive coping strategies, thereby inhibiting taking charge behaviors (Zahoor et al., 2024; Ahmad et al., 2025).

Conversely, when employees possess a low zero-sum mindset, the absence of an inherent, cutthroat mental model reduces their need for self-protection. As a result, AI benevolence effectively translates into psychological resources during the primary appraisal stage. This cue congruence between an employee’s non-competitive internal schema and the AI’s supportive cues, facilitates the secondary appraisal process, allowing employees to freely allocate their preserved cognitive bandwidth toward metacognitive reflection (Paek and Kakkar, 2025; De Boeck and Liu, 2026). Under these favorable cognitive conditions, metacognitive strategies function as a critical mechanism, converting the psychological safety afforded by AI into actionable insights for improvement. Ultimately, by activating a problem-focused coping mechanism, this process fosters employees’ taking charge aimed at optimizing the broader work system (Zhu and Li, 2025; Qiao et al., 2026).

*H5*: Zero-Sum Mindset moderated the indirect effect of AI benevolence and taking charge via metacognitive strategies, such the indirect effect was weaker in high zero-sum mindset.

## 3 Methods

### 3.1 Participants and procedure

The present study selected applied business and creative roles (e.g., content operations, product managers, and UI designers) within the technology sector as the research sample for two primary reasons (Dennis et al., 2023; Narayanan et al., 2026; Ali et al., 2026). First, unlike backend algorithm developers, employees in these applied roles frequently collaborate with intelligent assistive systems (e.g., GenAI). Consequently, they are more sensitive to the inclusivity and support offered by these systems (i.e., AI benevolence). Such perceptions are critical for enabling these employees to allocate liberated cognitive bandwidth toward reflecting on and reengineering business workflows (i.e., metacognitive strategies and taking charge), thereby facilitating their transition from routine executors to strategic thinkers. Second, these employees, who rely heavily on creative ideation and process optimization, operate within the technology industry’s hyper-competitive ecosystem, characterized by high turnover rates and intense market competition. This unique context provides an ideal empirical setting to examine the interaction between perceived AI benevolence and employees’ zero-sum mindset.

Guided by these two conditions, we evaluated an initial pool of eight prospective organizations and ultimately selected six internet companies that strictly met our research requirements. Assisted by their HR directors, we obtained employee rosters and distributed online surveys via email. To reduce common method bias, we used a temporal separation approach, collecting data at three time points with two-week intervals (Podsakoff et al., 2012). Confidential employee IDs were used as matching codes to link responses across waves without compromising anonymity. At Time 1, participants were asked to report their age, organizational tenure, gender, and education level, as well as provide data on AI benevolence and Zero-sum mindset; during this phase, 393 emails with a questionnaire link were distributed, and 366 valid responses were collected. Two weeks later at Time 2, the same group of participants was invited to complete questionnaires measuring metacognitive strategies, with 340 participants finishing this second-stage survey. Another 2 weeks later at Time 3, participants were asked to provide information on taking charge, and 317 completed this third-stage questionnaire. After excluding questionnaires that failed to meet pre-defined quality criteria (e.g., incomplete responses, inconsistent answers), the final effective sample consisted of 313 employees from 6 different internet companies (participants from each company ranged from 21 to 79), yielding an overall response rate of 79.64%. The demographic and occupational characteristics of the final sample were as follows: ages ranged from 22 to 55 years (M = 34.24, SD = 7.27), organizational tenure spanned 1 to 35 years, 50.50% of participants were male, and 235 participants (75.08%) held a bachelor’s degree.

### 3.2 Measures

All measurement instruments were translated into Chinese using the translation-back-translation procedure. With the exception of demographic variables and zero-sum mindset, all items were rated on a 5-point Likert scale, where responses ranged from 1 (“strongly disagree”) to 5 (“strongly agree”). AI Benevolence was measured at T1 using a three-item scale from Shi et al. (2021). AI Benevolence (*α* = 0.76) included an item such as “When assisting me with my work, the AI system will fully consider results that are beneficial to me personally.” Metacognitive strategies were measured at T2 using a four-item scale from Sun et al. (2025). Metacognitive strategies (*α* = 0.82) included an item such as “I kept thinking about the specific strategies I could use to perform the task well.” Taking charge was measured at T3 using a four-item scale from Parker and Collins (2010). Taking charge (*α* = 0.75) included an item such as “I try to bring about improved procedures in my workplace.” The zero-sum mindset included an item: “How my organization viewed success on a scale ranging from 1 ‘wealth can grow so there is enough for everyone’ to 10 ‘people can only get rich at the expense of others’” (Wood et al., 2008). Control variables. We controlled for the effects of demographic variables, including gender (1 = male, 2 = female), age (in years), tenure (in years), and education (1 = high school or below, 2 = junior college, 3 = bachelor, 4 = master or above), given their potential influence on taking charge (Qiao et al., 2026).

## 4 Results

### 4.1 Common method bias analysis

Following the recommendation of Podsakoff et al. (2003), Harman’s single-factor test was used to test for common method variance. The variance interpretation rate of the first factor was 39.80% (lower than 40% and below half of the total variance explained), which meant that no severe common method variance could be observed. An unmeasured latent method construct approach was employed, adding a latent common method factor to the three-factor model to construct a four-factor model. The results indicated that the fit indices of the model with the latent common method factor (χ^2^/df = 1.11, RMSEA = 0.019, CFI = 0.998, TLI = 0.995, SRMR = 0.026, Table 1) were not significantly better than those of the original model, suggesting that common method bias (CMB) wasn’t a severe issue in this study.

**Table 1:** Result of confirmatory factor analysis.

Model	Factor	χ^2^/df	RMSEA	CFI	TLI	SRMR
Three-factor model	AIB, MS, TC	1.47	0.039	0.985	0.979	0.038
Two-factor model ^a^	AIB + MS, TC	4.59	0.107	0.880	0.841	0.072
Two-factor model ^b^	AIB, MS + TC	5.55	0.121	0.848	0.799	0.087
One-factor model	AIB, MS, TC	8.69	0.157	0.736	0.660	0.109
Unmeasured latent method factor model		1.11	0.019	0.998	0.995	0.026

AIB, AI Benevolence; MS, Metacognitive strategies; TC, Taking charge. ^a^AIB and MS were combined into a single factor. ^b^MS and TC were combined into a single factor.

### 4.2 Descriptive statistics

Means, standard deviations, and correlations are presented in Table 2. As shown in Table 2, AIB was positively related to MS (*r* = 0.500, *p* < 0.01). MS was positively related to TC (*r* = 0.393, *p* < 0.01).

**Table 2:** Means, standard deviations (SD), and correlations of study variables.

Construct	1	2	3	**4**	5	6	7	8
1 AIB	(0.74)							
2 MS	0.500^**^	(0.79)						
3 TC	0.199^**^	0.393^**^	(0.74)					
4 ZM	0.021	0.041	−0.088	--				
5 Age	−0.074	−0.067	0.097	−0.111^*^	--			
6 Gender	−0.131^*^	−0.016	0.002	−0.089	0.021	--		
7 Edu	0.093	0.183^**^	0.023	0.112^*^	−0.057	−0.022	--	
8 Tenure	−0.080	−0.096	0.088	−0.132^*^	0.965^**^	0.030	−0.172^**^	--
Mean	3.62	3.81	3.80	4.08	34.24	1.50	2.78	11.13
SD	0.64	0.66	0.58	2.21	7.27	0.50	0.66	7.29

*N* = 313; ^**^p < 0.01; ^*^p < 0.05; () = the square root AVE.

### 4.3 Hypotheses testing

#### 4.3.1 Mediating effect test

H1 proposed that AIB was positively related to MS. As presented in Figure 1, the regression coefficient of AIB on MS was positive and significant (*β* = 0.478, *p* < 0.001), supporting H1. MS was positively and significantly related to TC (*β* = 0.358, *p* < 0.001), thus H2 was supported. To test the mediation effects of AIB on TC via MS, we computed the confidence intervals of the indirect links and conditional indirect links by using bootstrapping with 5,000 replications (Preacher et al., 2007). As depicted in Table 3, the mediation analysis indicates that the bootstrapped 95% confidence interval of the indirect effect of AIB on TC via MS excludes 0 (indirect effect = 0.183, 95% CI = [0.108, 0.262]). Thus, H3 was supported.

**Figure 1:**
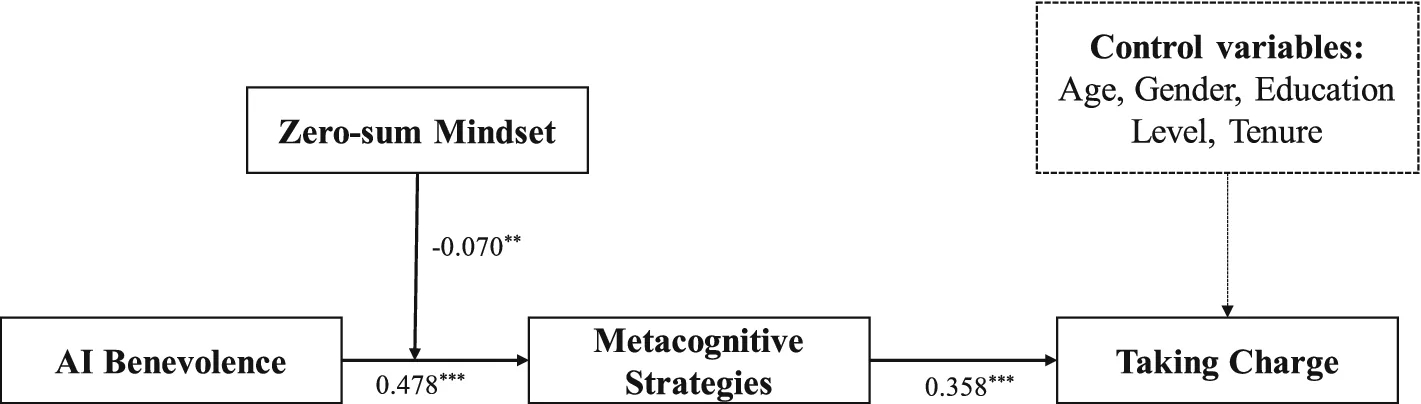
Path analysis results of the overall theoretical framework.

**Table 3:** Results of mediating effect test.

Path	Estimate	S. E.	95% CI
Indirect effect (AIB → MS → TC)	0.183	0.039	[0.108, 0.262]
Direct effect	0.010	0.056	[−0.100, 0.120]
Total effect	0.191	0.052	[0.091, 0.295]

#### 4.3.2 Moderating effect test

H4 proposed that ZM moderated the relationship between AIB and MS. The interaction term of AIB and ZM on MS is negative and significant (γ = −0.070, *p* < 0.01). Furthermore, to clearly interpret the significant interactions, we followed the recommendation of Preacher et al. (2007) to examine the moderating effects by testing the simple slopes at high and low levels of ZM, and the moderating effect diagram is drawn according to the regression coefficient (Figure 2). As evidenced by Figure 2, the slope depicting the relationship between AIB and MS is markedly steeper under conditions of low ZM (simple slope = 0.632, SE = 0.064, *p* < 0.001) compared to high ZM (simple slope = 0.323, SE = 0.079, *p* < 0.001). This pronounced gradient indicates a significantly weaker and less dynamic association between AIB and MS when ZM is higher. Thus, H4 was supported.

**Figure 2:**
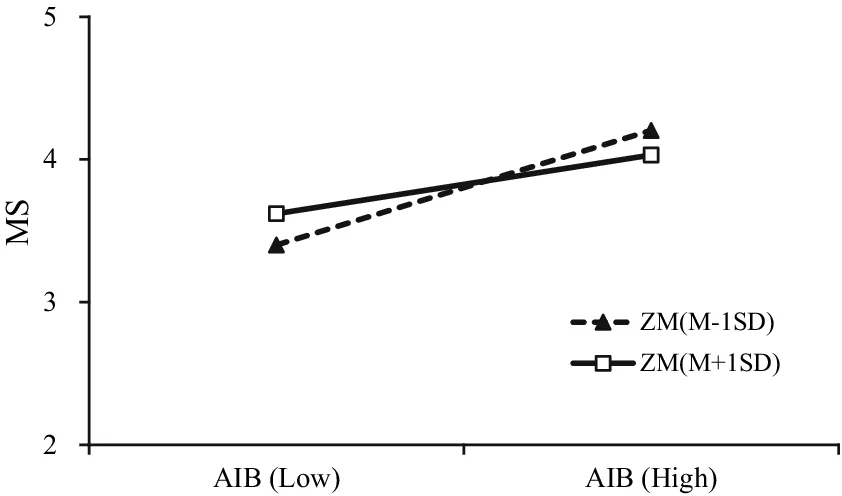
Moderating effect of ZM on the relationship between AIB and MS.

To establish the conditional indirect links, we calculated the index of moderated mediation (Hayes, 2015). H5 addressed the conditional indirect link between AIB and TC via MS. As the index of moderated mediation was significant and excluded 0 (index = −0.025, 95%CI [−0.048, −0.007]), H5 was supported (detailed results of the conditional indirect effect can be found in Table 4).

**Table 4:** Results of moderated mediating effect test.

Path	ZM	Estimate	S. E.	Boot 95%CI
AIB → MS → TC	High (+1 SD)	0.116	0.040	[0.040, 0.191]
	Low (−1 SD)	0.226	0.048	[0.138, 0.326]
	Differences	−0.025	0.010	[−0.048, −0.007]

#### 4.3.3 Post-hoc analysis

Given that our sample comprises employees drawn from different organizations, unobserved heterogeneity, such as variations in corporate culture, AI resources, or HR policies, might confound our findings (Antonakis et al., 2010). To rigorously rule out these potential organizational effects and verify the stability of our model, we conducted a supplementary robustness check by incorporating organizational fixed effects. Specifically, we generated dummy variables for the sampled organizations and included them as additional control variables in all primary estimation models. The results indicated that AIB remained positively related to MS (*β* = 0.481, *p* < 0.001), and MS remained positively related to TC (*β* = 0.358, *p* < 0.001). Furthermore, the interactive effect of AIB and ZM on MS was negative and significant (γ = −0.078, *p* < 0.001). Overall, because the direction, magnitude, and statistical significance of all hypothesized paths remained largely unchanged, we conclude that our theoretical model is robust.

## 5 Discussion

### 5.1 Theoretical contributions

Situated in the context of the digital workplace, this study draws on CAT to conceptualize and empirically examine how and when AI benevolence triggers employees’ taking charge behavior. In doing so, we make the following contributions to the organizational behavior and human-AI collaboration literature. First, we advance human-AI collaboration research by elucidating the specific relational characteristics of AI that empower employees to challenge the status quo. While recent scholarship has increasingly recognized the positive outcomes of AI integration (e.g., job crafting, human-AI collaboration), the literature still lacks a precise theoretical explanation for what specific AI attributes provide the psychological resources necessary for high-risk, proactive change (Zhang et al., 2026; Ma et al., 2026). Our study addresses this by introducing AI benevolence as a distinct, supportive relational construct. Specifically, we move beyond the generic concept of AI anthropomorphism—which merely serves as a prerequisite for social interaction—and distinguish AI benevolence from basic AI warmth or empathy. By conceptualizing AI benevolence as an actionable goodwill characterized by non-judgmental and error-tolerant support, we demonstrate that it functions as a crucial relational resource. This unique characteristic provides the essential psychological safety, devoid of human-like evaluation apprehension, that is required for individuals to engage in risky, constructive efforts to reconfigure workflows and initiate organizational change (i.e., taking charge).

Second, we enrich CAT by identifying metacognitive strategies as the crucial higher-order cognitive pathway that translates perceived AI benevolence into taking charge. Prior research exploring the behavioral outcomes of AI characteristics has predominantly emphasized affective responses (e.g., satisfaction and trust) or workload metrics, leaving higher-order cognitive mechanisms largely underexplored (Lin et al., 2024; Dong et al., 2026). Drawing on the primary and secondary appraisal mechanisms inherent in CAT, we theorize how AI benevolence fosters positive cognitive appraisals and frees up cognitive bandwidth, thereby cultivating metacognitive strategies (i.e., “thinking about thinking”). These strategies, in turn, equip employees with the cognitive acuity to discern systemic inefficiencies and opportunities for change while facilitating problem-focused coping behaviors, ultimately stimulating taking charge (Ahmad et al., 2025; Montefiore et al., 2026).

Finally, we expand the theoretical boundaries of CAT by identifying an individual’s zero-sum mindset as a crucial cognitive boundary condition for human-AI interaction. Although prior research has largely focused on general individual traits or leadership factors as moderators, the constraining influence of employees’ deep beliefs regarding resource scarcity remains underexplored (Agarwal et al., 2026; Shen et al., 2026). Drawing on CAT, we posit that when employees harbor a strong zero-sum mindset, believing that workplace success is strictly a win-lose game, the positive signals of AI benevolence are overshadowed by their chronic internal threat appraisals. Under such conditions, the non-judgmental support exhibited by AI creates a significant cue conflict with the employee’s internal hypercompetitive schema. Consistent with CAT’s primary appraisal mechanism, individuals with this defensive mental model are predisposed to prioritize threat detection (Paek and Kakkar, 2025; Dong et al., 2026). Consequently, even if the AI itself poses no direct harm, these employees deplete their limited cognitive resources by guarding against perceived coworker competition and anticipated resource loss. By elucidating how this internal cognitive interference ultimately attenuates the beneficial effects of AI benevolence on metacognitive strategies and subsequent proactive behaviors, our study provides a more nuanced understanding of how individual schemas shape human-AI collaboration dynamics.

### 5.2 Managerial implications

As generative AI becomes increasingly prevalent, organizations naturally tend to focus on its efficiency and cost-reduction capabilities (Zhang and Liu, 2026; He et al., 2026). However, our findings offer practical implications for those seeking to foster employee proactivity and enhance synergistic human-AI collaboration. First, expanding the focus beyond purely technical specifications to intentionally incorporate benevolent features during AI procurement and implementation provides a significant strategic advantage. Our results suggest that AI benevolence acts as a catalyst, encouraging employees to constructively challenge the status quo and engage in taking-charge behaviors. Consequently, successful integration entails balancing algorithmic precision with the affective cues present in human-AI interactions. Specifically, prioritizing AI systems with nonjudgmental interfaces that offer constructive feedback and tolerate user errors effectively supports this balance (Shi et al., 2021; Xu et al., 2026). Concurrently, carefully framing internal communications effectively mitigates potential anxieties surrounding AI adoption. Emphasizing that AI functions as a supportive resource, rather than a competitor, facilitates a positive and productive digital transition.

Second, expanding talent development beyond basic operational skills to cultivate higher-order metacognition offers significant organizational value. Our findings highlight metacognitive strategies as a key mechanism translating human-AI interactions into taking charge. As AI automates routine tasks, employee value increasingly lies in the capacity to reflect upon and optimize workflows, rather than merely maintaining software proficiency (Sun et al., 2025; Ali et al., 2026). Consequently, updating training frameworks to support this shift provides a strategic advantage for human resource departments. For instance, allocating dedicated time for reflection enables employees to periodically step back from daily routines and identify opportunities for enhancing human-AI collaboration. Furthermore, coupling these practices with targeted incentive mechanisms, such as formally recognizing and rewarding employees who proactively design alternative workflows, effectively reinforces such proactive behaviors. Ultimately, fostering this reflective capacity mitigates passive compliance, instead empowering employees to actively and constructively shape their digital work environments (Gabriel et al., 2022; Dennis et al., 2023).

Finally, organizations are strongly encouraged to mitigate employees’ zero-sum mindset, which often constrains the beneficial effects of AI benevolence. Our findings indicate that when individuals hold a highly competitive cognitive schema (i.e., a high zero-sum mindset), the advantages of benevolent AI are significantly attenuated, as employees tend to divert their cognitive resources toward interpersonal threat appraisals. Consequently, technological empowerment initiatives frequently yield limited results without corresponding HR interventions to reshape these subjective mindsets (Dong et al., 2023; Paek and Kakkar, 2025). For instance, when AI investments are paired with hyper-competitive performance systems, such as forced distribution or zero-sum reward pools, employees’ cognitive schemas are skewed toward peer defensiveness. To maximize AI efficacy, managers would benefit from reassessing human resource practices that inadvertently foster internal competition. Gradually shifting toward absolute performance evaluations, integrating team-level collaborative metrics, and cultivating a culture focused on mutual gains offers substantial organizational value. Ultimately, reshaping employees’ competitive schemas to align with AI benevolence, achieving a “win-win” cognitive congruence, remains key to translating technological investments into proactive, taking charge.

### 5.3 Limitations and future directions

This study has several limitations that future research can address. First, although we attempted to reduce common method bias (CMB) by measuring constructs at three distinct time points (Podsakoff et al., 2003), our data were self-reported and derived from a single source. Future studies can address the CMB issue more effectively by collecting data from multiple sources. Second, our non-longitudinal design hinders drawing causal inferences. Future studies may adopt more rigorous longitudinal designs (e.g., cross-lagged panel designs) to validate our findings. Third, the measurement of the zero-sum mindset relied on a single-item indicator, which poses inevitable limitations regarding construct validity and the generalizability of our findings. Although this single item adequately captures employees’ subjective perceptions within our research context, it may not fully reflect the complexity of the zero-sum construct. Future studies are encouraged to develop or adopt multi-item scales to enhance measurement reliability and validity. Finally, this study was conducted in a single industry (Internet Company), which limits the generalizability of our results. Considering the importance of AI Benevolence for employees’ taking charge and the differences across various industries, testing our results in other industries would be both meaningful and valuable.

## 6 Conclusion

Situated in the context of the digital workplace, this study draws on CAT to conceptualize and empirically examine how and when AI benevolence stimulates employees’ taking charge behaviors. Results from a three-wave survey of employees in non-technical yet highly collaborative roles (e.g., product management, operations, and design) within the technology sector reveal that: (1) metacognitive strategies mediated the relationship between AI benevolence and taking charge; and (2) a zero-sum mindset not only attenuated the positive effect of AI benevolence on metacognitive strategies but also dampened the indirect effect of AI benevolence on taking charge via these strategies. Consequently, this study extends the literature on the antecedents of taking charge into the context of human-AI interaction, clarifying the mechanism through which AI benevolence cultivates higher-order cognition to foster taking charge. Furthermore, by demonstrating how human-AI dynamics are constrained by an overarching zero-sum mindset, this research establishes important boundary conditions. Ultimately, it offers a novel theoretical lens for organizational behavior research to understand how AI reshapes employee proactivity within digitally integrated workplaces.

## Data Availability

The raw data supporting the conclusions of this article will be made available by the authors, without undue reservation.
